# Supplementary material for: A Critical View of the Application of the APEX Software (Aqueous Photochemistry of Environmentally-Occurring Xenobiotics) to Predict Photoreaction Kinetics in Surface Freshwaters
Source: Molecules. 2019 Dec 18;25(1):9. doi: 10.3390/molecules25010009 (PMC7017383; doi:10.3390/molecules25010009)
Supplement: Supplementary file 1 [file molecules-25-00009-s001.zip › 3.2.4_gcc-4.4.0.zip!/3.2.4_gcc-4.4.0/3.2.4_gcc-4.4.0/doc/octave/html/interpreter/Using-Packages.html]

Using Packages - Untitled


Next: Administrating Packages,
Previous: Installing and Removing Packages,
Up: Packages


---

### 35.2 Using Packages

By default installed packages are available from the Octave prompt,
but it is possible to control this using the `pkg load` and
`pkg unload` commands. The functions from a package can be
removed from the Octave path by typing

```
     pkg unload package_name
```

where `package_name` is the name of the package to be removed
from the path.

In much the same way a package can be added to the Octave path by
typing

```
     pkg load package_name
```
